# The proteome of extracellular vesicles released by clastic cells differs based on their substrate

**DOI:** 10.1371/journal.pone.0219602

**Published:** 2019-07-10

**Authors:** Wellington J. Rody, Casey A. Chamberlain, Alyssa K. Emory-Carter, Kevin P. McHugh, Shannon M. Wallet, Victor Spicer, Oleg Krokhin, L. Shannon Holliday

**Affiliations:** 1 Department of Orthodontics and Pediatric Dentistry, Stony Brook University School of Dental Medicine, Stony Brook, NY, United States of America; 2 Department of Orthodontics, University of Florida College of Dentistry, Gainesville, FL, United States of America; 3 Department of Periodontology, University of Florida College of Dentistry, Gainesville, FL, United States of America; 4 School of Dental Medicine, East Carolina University, Greenville, NC, United States of America; 5 Manitoba Center for Proteomics and Systems Biology, Winnipeg, MB, Canada; 6 Department of Anatomy & Cell Biology, University of Florida College of Medicine, Gainesville, FL, United States of America; Charles P. Darby Children's Research Institute, UNITED STATES

## Abstract

Extracellular vesicles (EVs) from osteoclasts are important regulators in intercellular communication. Here, we investigated the proteome of EVs from clastic cells plated on plastic (clasts), bone (osteoclasts) and dentin (odontoclasts) by two-dimensional high performance liquid chromatography mass spectrometry seeking differences attributable to distinct mineralized matrices. A total of 1,952 proteins were identified. Of the 500 most abundant proteins in EVs, osteoclast and odontoclast EVs were 83.3% identical, while clasts shared 70.7% of the proteins with osteoclasts and 74.2% of proteins with odontoclasts. For each protein, the differences between the total ion count values were mapped to an expression ratio histogram (Z-score) in order to detect proteins differentially expressed. Stabilin-1 and macrophage mannose receptor-1 were significantly-enriched in EVs from odontoclasts compared with osteoclasts (Z = 2.45, Z = 3.34) and clasts (Z = 13.86, Z = 1.81) and were abundant in odontoclast EVs. Numerous less abundant proteins were differentially-enriched. Subunits of known protein complexes were abundant in clastic EVs, and were present at levels consistent with them being in assembled protein complexes. These included the proteasome, COP1, COP9, the T complex and a novel sub-complex of vacuolar H^+^-ATPase (V-ATPase), which included the (pro) renin receptor. The (pro) renin receptor was immunoprecipitated using an anti-E-subunit antibody from detergent-solubilized EVs, supporting the idea that the V-ATPase subunits present were in the same protein complex. We conclude that the protein composition of EVs released by clastic cells changes based on the substrate. Clastic EVs are enriched in various protein complexes including a previously undescribed V-ATPase sub-complex.

## Introduction

Extracellular vesicles (EVs) released by osteoclasts are important regulators of bone remodeling [1–4]. RANK-containing EVs from osteoclasts regulate osteoclastogenesis by a paracrine mechanism [1]. Very recently, RANK-containing EVs released by osteoclasts were found to bind osteoblasts through RANKL [4;5]. This binding stimulated RANKL reverse signaling in osteoblasts through the Runt-related transcription factor 2 (Runx2) pathway. This led to increased osteoblast differentiation *in vitro* and increased bone formation *in vivo*. It was postulated that RANK-containing EVs released by osteoclasts are vital for coupling bone resorption with bone formation [4].

We recently showed by low resolution one-dimensional mass spectrometry that there are differences in the protein composition of EVs released by osteoclasts (clastic cells resorbing bone) and odontoclasts (clastic cells resorbing dentin) [6]. Clastic cells are multinuclear cells responsible for the physiological and pathological breakdown of mineralized tissues, including bone and tooth dentin. While the consensus on the nomenclature for these ‘clastic’ cell types is still evolving, some authors propose to name them based on the matrix that they resorb [7–10]. Osteoclasts and odontoclasts are thought to differentiate from the same hematopoietic stem cells, but little is known about the timing and mechanism of their differentiation [7]. Key differences in odontoclastic and osteoclastic activity have been reported. Odontoclasts are not regulated by parathyroid hormone (PTH) as are osteoclasts [11]. Moreover, odontoclasts show higher activity in forming resorption lacunae, and interleukin-1 (IL-1) stimulates the formation of resorption lacunae on dentin to a higher extent than bone [12]. Therefore, under specific circumstances, osteoclasts and odontoclasts may behave and function differently.

EVs are 30–150 nm in diameter [13]. They include exosomes, which are derived from multivesicular bodies, and microvesicles, which bud directly from the plasma membrane [13]. EVs carry proteins, lipids, metabolites and ribonucleic acids (RNAs), and can transmit these elements to target cells. They play key roles in intercellular communication and signaling and they show promise as biomarkers and as vehicles for drug delivery [13–15]. EVs are present in most biological fluids, including blood, urine, saliva, mucus, breast milk, bile, and gingival crevicular fluid [14,16,17].

To gain greater insight into the composition of EVs from clastic cells, we performed two-dimensional high performance liquid chromatography mass spectrometry (2D HPLC-MS/MS) on EVs from clasts on plastic, odontoclasts and osteoclasts. Our data confirm that the substrate regulates the protein composition of the EVs released by clastic cells. We find evidence for numerous protein complexes, including a novel V-ATPase sub-complex, which make up a large component of the total protein composition of clastic EVs. Differences in the protein composition of osteoclasts and odontoclasts that are identified provide a basis for studying the regulatory mechanisms of clastic EVs and identify potential biomarkers for pathological dental root resorption and other oral diseases.

## Materials and methods

### Experimental design and cell culture protocol

**Fig 1:**
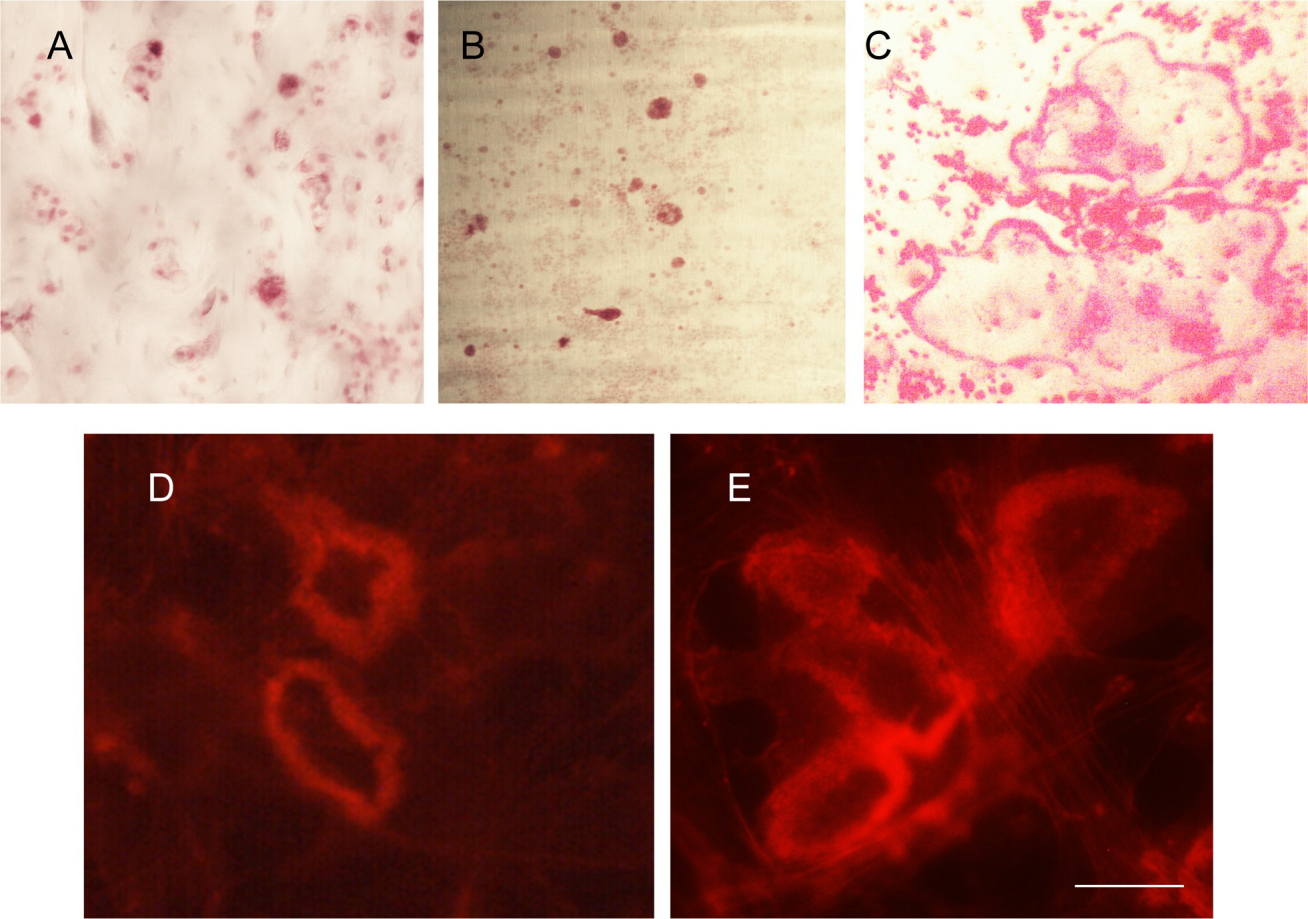
Generation of osteoclasts, odontoclasts and clasts in cell culture. Presence of tartrate-resistant acid phosphatase (TRAP) positive cells at the end of the cell culture period on (A) osteoclasts on bone slice; (B) odontoclasts on dentin slice; (C) clasts on plastic tissue culture plate. All photos are at the same magnification. Note that on plastic some giant cells with more than 10 nuclei form whereas on bone or dentin rarely are cells with more than 5 nuclei detected. To confirm that osteoclasts (D) and odontoclasts (E) were resorbing, actin rings, which are surrogate markers for resorbing osteoclasts and odontoclasts, were stained with rhodamine-phalloidin. The scale bar is 66 μm in A-C and 12 μm in D and E.

The use of animals for this study was approved by the University of Florida Institutional Care and Use Committee (IACUC, protocol number: 201207596) and carried out in accordance with the recommendations in the guide for care and use of laboratory animals of the National Institutes of Health. No procedure involving pain or suffering were performed on the mice prior to their sacrifice. Briefly, clastic cells were grown as described in detail in previous publications [1,6]. Precursors were isolated from the bone marrow of femur and tibiae of 4 to 6 week-old wild type C57Bl/6 mice by flushing with alpha-MEM (2.5 ml/bone) using a 10-ml syringe and 25-gauge needle. The mononuclear cells were then plated at a density of 0.5 x 10^6^ cells/well on bone slices, dentin discs or empty plastic tissue culture wells (Fig 1A) and cultured for seven days with 10% EV-depleted fetal bovine serum (Exo-FBS, System Biosciences). To induce osteoclastogenesis, mononuclear cells were supplemented with recombinant mouse M-CSF (20 ng/ml; R&D Sys.) and recombinant soluble mouse RANKL (20 ng/ml; R&D Sys.). Medium was changed twice after the viability of the cells was confirmed by visual inspection using a light microscope. At the end of the cell culture period, clastic cell formation was assessed by staining for tartrate-resistant acid phosphatase (TRAP) using a leukocyte phosphatase kit (Sigma-Aldrich) as per the manufacturer’s instruction [18]. Resorption status was determined by using actin ring-formation as a surrogate marker for bone resorption as previously described [19]. Experiments were performed in triplicate to reduce random error and experimental bias.

### EV isolation and quantitation

EVs were isolated as described previously [1,6]. Cell conditioned media from culture period Days 4–7 was removed from the wells, pooled in sterile 15 ml conical tubes and centrifuged at 3,000g for 30 minutes at room temperature. The supernatant was transferred to new tubes and ExoQuick TC solution (System Biosciences) was added to the media at a ratio of 1:5. The samples were incubated overnight at 4°C then centrifuged at 1,500g for 30 minutes to remove the supernatant. The pellets were resuspended in 150 μl PBS, subjected to ultracentrifugation at 100 K x *g* using in an Airfuge (Beckman), and the pellets were frozen at -80°C for future analyses. EVs were quantified in 10 μL of the resuspended pellet by measuring the enzymatic activity of acetecylcholinesterase (AChE) using the EXOCET Quantitation kit (System Bioscience) per the manufacturer’s instructions. We have found that the estimates of EV numbers obtained by EXOCET agreed closely with numbers obtained by nanoparticle tracking using a NanoSight NS300 (Malvern).

### Proteomics profiling

EVs from osteoclasts, odontoclasts and non-resorbing clastic cells (cells on plastic) were pooled across three rounds of experiments for two dimensional high performance liquid chromatography-mass spectrometry analysis (2D HPLC-MS/MS). The isolated EVs were solubilized in 1 M urea/0.2 M Tris/HCl buffer pH 7.6, and the proteins digested with trypsin using the Filter Aided Sample Preparation (FASP) technique [20]. Resulting digests were acidified with trifluoroacetic acid (TFA) and purified by reversed-phase solid-phase extraction. Each sample contained 5–10 μg of digested EV proteins as determined by Nanodrop 2000 (Thermo Fisher Scientific, Rockford IL). The 2D HPLC-MS/MS analysis of the EV extracts was performed as described in detail previously [21]. Agilent 1100 series LC system with UV detector (214 nm) and 1mm×100mm XTerra C18, 5 μm column (Waters, Ireland) was used for pH 10 first dimension reversed-phase separation [21]. 1.25% acetonitrile per minute gradient (0–40% acetonitrile in 32 min) was delivered at 150 μL/min flow rate. Both eluents A (water) and B (1:9 water:acetonitrile) contained 20 mM ammonium formate pH 10. Thirty 1-min fractions were collected and concatenated into 10 to provide optimal separation orthogonality [21]. Second dimension LC-MS/MS has been performed using a nano LC-MS system coupled to a Triple TOF 5600 mass spectrometer (ABSciex, Toronto, Ontario, Canada), via an IonSpray III nano-source (ABSciex). A splitless nano-flow 2D LC Ultra system (Eksigent, Dublin, CA) was used to deliver water/acetonitrile gradient at 500 nL/min flow rate through a 100μm×200mm analytical column packed with 3μm Luna C18(2) (Phenomenex, Torrance, CA). Sample injection for individual fractions via a 300μm×5mm PepMap100 trap-column (Thermo Fisher Scientific) was used in all experiments. The gradient program included following steps: linear increase from 0.5 to 30% of buffer B (acetonitrile) in 78 min, 5 min columns wash with 90% B and 8 min system equilibration using starting conditions of 0.5% B (0.38% acetonitrile per minute gradient, 90 min total run time). Both eluents A (water) and B (acetonitrile) contained 0.1% formic acid as ion-pairing modifier. Each Triple TOF 5600’s measurement cycle consisted of a 250-ms MS measurement (m/z values of eluting peptides) and up to 20 MS/MS (100 ms each) performed on the most abundant peaks. Ten individual concatenated fractions from the first separation dimensions were analyzed for each sample. The resulting 10 mascot generic format (MGF) spectra files were sequentially concatenated into a single file for peptide identification and quantitation, which was conducted in-house at the Manitoba Center for Proteomics (Winnipeg MB, Canada) using X!Tandem search algorithm and additional retention time prediction filtering [22,23] with 20 parts per million (ppm) and 50 ppm mass tolerance for parent and fragment ions, respectively. Additional X!Tandem parameters included: oxidation of Met, Trp; N-terminal cyclization at Gln, Qln, Cys; N-terminal acetylation, phosphorylation (Ser, Thr, Tyr); deamidation (Asn and Gln); an expectation value cut-off of log(e)<−1 for both proteins and peptides. Identified single-missed-cleavage tryptic peptides from the *Mus musculus* Uniprot database (16,704 proteins) were quantified using the sum of their MS2 fragment intensities.

### Statistical and data analysis of mass spectrometry results

Each protein quantitation entry was the log2 transform of the sum of the intensities of its member peptides, with a rule of a peptide minimum expectation value log(e)< = -1.5 each, and at least two non-redundant peptides per protein required for quantitation. Protein-level differences between samples were then mapped into Z-scores, i.e. the distance from the population mean in units of standard deviation. Z-scores were calculated for three pairwise comparisons as follows: 1) odontoclasts versus non-resorbing clasts, 2) osteoclasts versus non-resorbing clasts, and 3) osteoclasts versus odontoclasts. Each comparison gave a three-mode population, with proteins quantified in one state but not the other forming the negative and positive outer sets respectively, and proteins quantified in both states forming the central population. The mean and standard deviation of this central population was computed and used to transform the entire set on a protein-by-protein level into Z-scores using the equation: Z-score = (Z—mean) / SD. Proteins with Z-scores greater than 1.65 or smaller than -1.65 (the outermost 10% of the distribution) were considered significantly higher or lower in one EV sample versus another.

### Detection of protein complexes

Inspection of the proteomic data revealed that subunits of stable protein complexes with specific stoichiometry were abundant in samples. These appeared in similar ratio’s that were consistent in various samples. To better understand these complexes, we plotted them based on the molecular weight of the components and expected stoichiometry of the complex versus their relative abundance in EVs estimated as described above.

### Immunoprecipitation of V-ATPase sub-complex

High affinity protein A-agarose (Abcam) was incubated with rabbit antisera anti-E V-ATPase subunit. The antibody was crosslinked to protein A-agarose using DMP (Sigma) as we have described [24]. EVs from three-day conditioned media from one 100 cm tissue culture plate were homogenized in 200–500 μl solubilization buffer (SB; 20 mM Tris/HCl pH 7.4, 5 mM Sodium Azide, 1 mM EDTA, 1 mM dithiothreitol, 1.5% n-octyl β glucopyrannoside, 0.6% CHAPS, 10% glycerol plus PMSF, and a cocktail of addition protease inhibitors). EVs were then homogenized by 15 passes in a 1 ml, tight fitting, glass homogenizer. Samples were incubated with 15 μl of the antibody conjugated protein A beads for 2 hours at 4 degrees Celsius, then washed 3X in 0.5 ml ice cold NET-gel buffer (50 mMTris/HCl pH 7.4, 150 mM NaCl, 0.1% Nonidet P-40, 1 mM EDTA, 0.25% gelatin, 5 mM NaAzide). The beads were then resuspended in 30 μl SDS-PAGE sample buffer, heated to 95 degrees Celsius, and the soluble material was separated by SDS-PAGE, blotted to nitrocellulose and probed with anti-PRR antibody (AbCam).

### Use of proteomic data tools

Gene ontology and biological process enrichment analysis of the EV proteins that were uniquely found in one matrix versus the other was carried out using FunRich (www.funrich.org), an open access functional enrichment analysis tool.

## Results

### EV preparation from clasts, osteoclasts and odontoclasts

Clastic cells precursors were differentiated on plastic (clasts), bone (osteoclasts), and dentin (odontoclasts). Multinuclear TRAP positive, clasts, osteoclasts and odontoclasts were detected after 7 Days (Fig 1A–1C). The resorbing activity of odontoclasts and osteoclasts was confirmed by staining cells with phalloidin to detect the distinctive actin ring structures that are characteristic of resorbing clastic cells (Fig 1D and 1E). EVs were isolated from conditioned media from clasts, osteoclasts and odontoclasts and EV numbers were determined by EXOCET quantitation (Systems Biosciences). Clasts produced the highest average number of EVs/well (9.72 x 10^9^), followed by osteoclasts (7.296 x 10^9^) and odontoclasts (5 x 10^9^).

**Fig 2:**
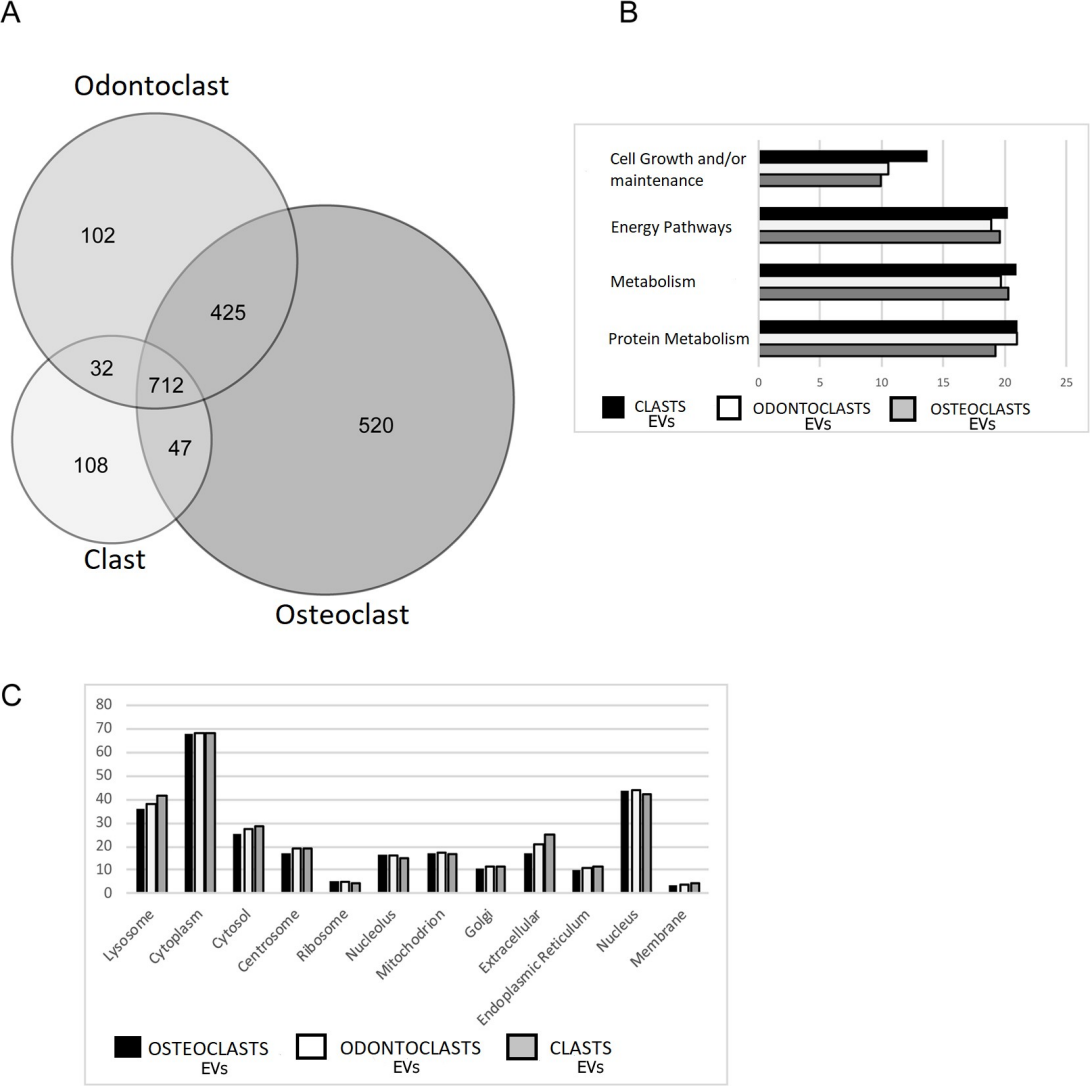
Overview of protein composition of EVs from clastic cells. (A) Venn diagram of proteins detected from osteoclasts, odontoclasts and clasts. (B) Comparison of the gene ontology biological pathways represented by 10% or more of the total proteins in EVs. (C) Selected primary cellular location of proteins detected in clastic EVs. These locations were abundant and indicative of the general feature that there was little difference between the groups of clastic EVs.

Mass Spectrometry analysis identified 1,952 proteins with log(e) scores <−10 and the presence of at least two peptides in the isolated EVs (S1 Table). Eight-four percent of these proteins (1640) were found in the ExoCarta database [25] confirming that there was an enrichment of exosomal proteins in our samples. Abundant proteins were even more likely to be found in ExoCarta. For example, of the 100 most abundant proteins in osteoclast EVs, 95 were in ExoCarta and two not found, TRAP type 5A and cathepsin K, are markers for clastic cells. A total of 102 proteins were found only in odontoclast EVs, whereas 524 unique proteins were identified in the EVs from osteoclasts. There was an overlap of 425 proteins between osteoclasts and odontoclasts, while 712 proteins were common among EVs from all clastic cells (Fig 2A). As a first approximation of the nature of the proteins detected, we sorted them into functional categories using FunRich (Fig 2B). Only four biological process categories were represented by 10% or more of the total proteins and there was little variation in the biological processes between EVs from different source cell-types. Proteins in EVs displayed a range of cellular locations, but there were no obvious differences between groups (Fig 2C). Comparing the 100 and 500 most abundant proteins found in each type of EV, osteoclasts and odontoclasts shared 79% and 83.3% of proteins, osteoclasts and clasts shared 71% and 70.7% and odontoclasts and clasts shared 74% and 74.2%.

### Statistical comparison of data sets

Pairwise comparisons at day 7 of the cell culture (osteoclasts *vs*. odontoclasts, osteoclasts *vs*. non-resorbing clasts, and odontoclasts *vs*. non-resorbing clasts) identified numerous proteins that were significantly enriched (Z > 1.65) and identified by at least 2 peptides in EVs from specific sources (S2 Table). Many of these were low abundance proteins that were identified by only 2 or 3 peptides. To better understand the proteins in EVs, we examined the nature and cellular location of the proteins detected.

**Fig 3:**
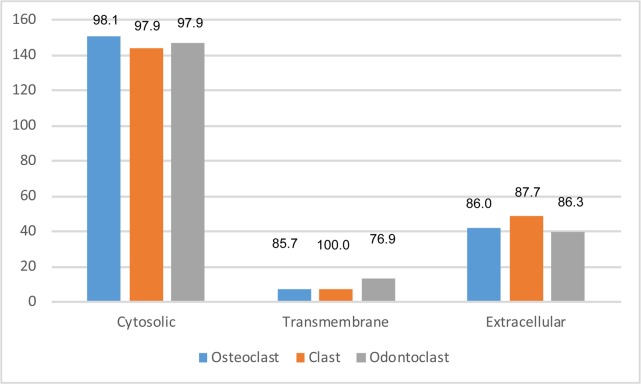
Categorization of proteins by cellular location. The 200 most abundant proteins in extracellular vesicles (EVs) were categorized based on their expected cellular location. Numbers at the top indicate the percentage of the proteins in the category reported to be extracellular vesicle (EV) proteins in ExoCarta. EVs from all types of clastic cells had mostly cytosolic proteins and very few transmembrane proteins.

Proteins are extracellular, transmembrane or cytosolic. We categorized the 200 most abundant proteins from clasts, osteoclasts and odontoclasts. Cytosolic proteins were most numerous, followed by extracellular proteins, and only a few transmembrane proteins were detected (Fig 3). There was also more variance among the samples with regard to abundant transmembrane proteins compared with cytosolic or extracellular proteins. Recent developments in sorting, isolating and characterizing EVs make transmembrane proteins attractive to use to detect and differentially isolate EVs [26]. We therefore focused on transmembrane proteins (Table 1). Our dataset from clastic cells was compared with published EV datasets from other cell types. These included human monocyte-derived macrophages (Mac), U87 glioblastoma cells (U87), Huh7 hepatocellular carcinoma cells (Huh7) and human bone marrow-derived mesenchymal stem cells (MSC) [27,28]. The presence of other cell types in parenthesis in the gene symbol column (Table 1) indicate that these proteins have been previously reported in EVs released by the indicated cell types.

**Table 1:** Selected transmembrane proteins in EVs with number of peptides and Z-scores.

Protein	Gene symbol (In EVs of indicated cells *)	Protein Description	# Peptides odontoclast	# Peptides osteoclasts	# Peptides clasts	Z-score Odontoclasts vs. clasts	Z-score Osteoclasts vs. clasts	Z-score Osteoclasts vs. odontoclasts
Q91ZX7	LRP1 (Huh7, Mac)	Prolow-density lipoprotein receptor-related protein 1	150	132	114	-0.17	-0.77	-0.95
Q62351	TFRC (Huh7, MSC, U87, Mac)	Transferrin receptor protein 1	29	31	29	-0.63	0.03	0.83
Q07113	IGF2R (Huh7, MSC, U87, Mac)	Cation-independent mannose-6-phosphate receptor	46	53	25	0.15	-0.53	-1.02
Q9CYN9	ATP6AP2 (MSC, U87, Mac)	(Pro) renin receptor (PRR)	13	16	9	0.21	0.43	0.33
Q60754	MARCO (Mac)	Macrophage receptor MARCO	6	5	-	13.99	11.58	-1.09
P43406	ITGAV (Huh7, MSC, U87, Mac)	Integrin alpha-V	13	36	20	-1.12	0.32	1.91
Q61207	PSAP (U87, Mac)	Prosaposin	20	22	8	1.61	0.69	-1.09
A2A8L5	PTPRF (Huh7)	Receptor-type tyrosine-protein phosphatase F	26	24	25	-0.93	-1.48	-1.02
Q2PZL6	FAT4	Protocadherin Fat 4	17	16	21	-0.99	-1.07	-0.32
P11835	ITGB2 (Mac)	Integrin beta-2	22	23	14	0.07	-0.5	-0.85
Q9Z1Q5	CLIC1 (Huh7, MSC, U87, Mac)	Chloride intracellular channel protein 1	11	11	9	0.12	-0.02	-0.22
Q8R4Y4	STAB1	Stabilin-1	63	38	-	13.86	10.54	-2.45
Q61830	MRC1 (Mac)	Macrophage mannose receptor 1	41	15	8	1.81	-0.65	-3.34
P05555	ITGAM (Mac)	Integrin alpha-M	20	19	11	0.6	0.31	-0.36
Q62465	VAT1 (U87, Mac)	Synaptic vesicle membrane protein VAT-1 homolog	11	12	3	1.13	0.51	-0.74
P35762	CD81 (Huh7, MSC, U87, Mac)	CD81 antigen	3	3	2	1.76	-0.05	-2.4
Q8R422	CD109 (U87, Mac)	CD109 antigen	4	29	-	9.98	11	3.26
Q08857	CD36 (MSC)	Platelet glycoprotein 4	2	-	-	9.89	-	-14.99
P40240	CD9 (Huh7, MSC, U87, Mac)	CD9 antigen	2	2	2	1.41	0.7	-0.84
P40237	CD82 (U87, Mac)	CD82 antigen	3	3	-	10.63	9.8	0.64
P15379	CD44 (U87, Mac)	CD44 antigen	4	5	4	-0.86	-0.01	1.07
O35305	TNFRSF11A	RANK	-	4	2	-10.71	-0.39	13.31
Q00651	ITGA4	Integrin alpha-4	4	3	-	9.96	7.56	-
P11688	ITGA5 (Huh7, MSC, U87, Mac)	Integrin alpha-5	-	2	-	-	7.34	12.15
Q62469	ITGA2 (Huh7, MSC, U87, Mac)	Integrin alpha-2	-	9	2	-12.14	-0.66	14.76
P09055	ITGB1 (Huh7, MSC, U87, Mac)	Integrin beta-1	11	11	10	-0.32	-1.01	-1.11
O54890	ITGB3 (Huh7, MSC, U87, Mac)	Integrin beta-3	7	20	9	-0.84	0.43	1.7
Q8BNA6	FAT3	Protocadherin Fat 3	4	-	3	-0.03	-10.27	-15.18
Q5F226	FAT2	Protocadherin Fat 2	-	-	2	-12.24	-11.27	-

*Parentheses after gene symbol indicates cell types where found: human monocyte-derived macrophages (Mac), U87 glioblastoma cells (U87), Huh7 hepatocellular carcinoma cells (Huh7) and human bone marrow-derived mesenchymal stem cells (MSC). Data from Huh7, MSC, U87 and Mac come from references [27,28].

A negative Z-score means that the protein was more abundant in extracellular vesicles from the second cell type in each pairwise comparison (Odontoclasts vs. Clasts, Osteoclasts vs. Clasts, Osteoclasts vs. Odontoclasts).

**Fig 4:**
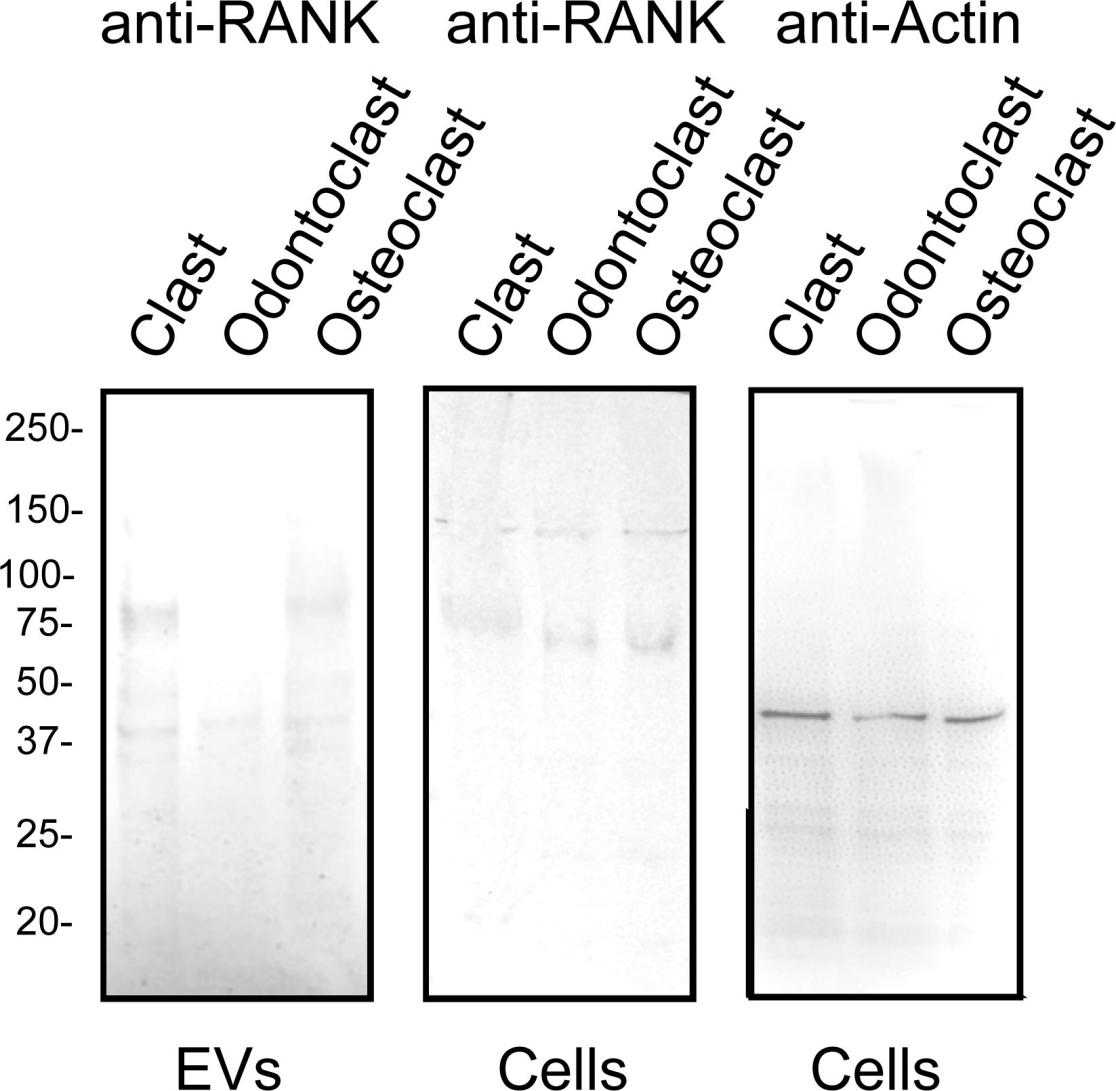
Immunoblot confirms that reduced levels of RANK are present in EVs from odontoclasts. *Left panel*; 3 X 10^7^ EVs (by nanoparticle tracking) isolated from clasts, odontoclasts and osteoclast were separated by SDS-PAGE, blotted to Immobilon P, and probed with anti-RANK antibody (Biorbyt, Cat # orb8560). Note the reduced level of RANK in the EVs from odontoclasts. *Middle panel*; 100 μg of protein from whole cell extracts of clasts, odontoclasts and osteoclasts was probed with the anti-RANK antibody. Levels of RANK were similar in all cell types. *Right panel*; The same cell extracts from clasts, odontoclasts and osteoclasts were probed with anti-actin (Sigma, Cat # A2066) to show that the loading of proteins was similar.

RANK may be both a component of biologically-active EVs and an EV-marker for diseases [1,4,29]. RANK was detected at low levels in clasts and slightly higher levels in osteoclasts, but was not detected in odontoclasts EVs (Table 1). Immunoblots confirmed that the level of RANK was reduced in EVs from odontoclasts. The level of RANK in the total cells extracts, however, was similar, suggesting that a mechanism to load RANK into EVs is repressed in odontoclasts compared to clasts and osteoclasts (Fig 4). Stabilin-1 was significantly more abundant in odontoclast EVs than osteoclast EVs (Z = - 2.45) and it was not detected in clasts (Z = 13.86). It therefore represents a promising potential biomarker for odontoclasts (Table 1). Macrophage mannose receptor-1 was somewhat less abundant, but was also significantly enriched in odontoclast EVs compared with osteoclast EVs (Z = - 3.34) and clasts (1.81) (Table 1).

We further examined various classes of transmembrane proteins. Cluster of differentiation (CD)81 and CD109 were found at relatively high levels in EVs from osteoclasts and odontoclasts and at low levels or not detected in clasts. CD36 was detected in odontoclasts, but not osteoclasts or clasts, making it another potential biomarker for root resorption in spite of its low abundance (Table 1). An abundant integrin detected in clastic EVs was alpha-M beta2. Three potential alpha partners, alpha 2, alpha 4 and alpha 5, for integrin Beta1 were found in clastic EVs. Only one potential partner for integrin Beta 3, alphaV, was detected at significantly higher levels (Z = 1.91) in EVs from osteoclasts compared with either odontoclasts or clasts (Table 1).

### Comparison of clastic cells with other cell types

Clastic cells are highly specialized to function on mineralized tissues. Thus, we wanted to compare the proteomic profile of clastic cells EVs to recently reported proteomic datasets of EVs from other cell types including human monocyte-derived macrophages (Mac), U87 glioblastoma cells (U87), Huh7 hepatocellular carcinoma cells (Huh7) and human bone marrow-derived mesenchymal stem cells (MSC) [27,28]. Some abundant complexes found in clastic EVs, such as the Arp2/3 complex, proteasome, COP1, COP9 and the T complex, were also present in EVs released by other cell types (Table 2). Unlike other V-ATPase elements, the isoforms of the a-subunit were not detected in clastic EVs. In contrast, the (pro) renin receptor (PRR), which was abundant in clastic EVs, was either not detected or was at very low levels in EVs from Mac, U87, Huh7 and MSC (Tables 1 and 2). Stabilin-1 was not detected in EVs from the cell types surveyed by previous authors, while macrophage mannose receptor-1 was detected in EVs from macrophages (Table 1). RANK was not detected in EVs from other sources (Table 1).

**Table 2:** Protein complexes found in clastic cells and other cell types.

**Coatamer protein 1 (COP1)**	**T-Complex**	**20S Proteasome alpha subunits**	**20S Proteasome Beta subunits**	**26S proteasome Regulatory subunits**
***cops8*** (U87, Mac) ***cops7a*** (MSC, U87, Mac) cops7b (Huh7, MSC, U87) cops5 (Huh7, MSC, U87, Mac) cops6 (U87, Mac) cops3 (Huh7, MSC, U87, Mac) cops4 (Huh7, MSC, U87, Mac) ***cops2*** (Huh7, MSC, U87, Mac) gps1 (Huh7, MSC, U87, Mac)	CCT2 (Huh7, MSC, U87, Mac) CCT8 (Huh7, MSC, U87, Mac) CCT4 (Huh7, MSC, U87, Mac) CCT6A (Huh7, MSC, U87, Mac) CCT7 (Huh7, MSC, U87, Mac) CCT5 (Huh7, MSC, U87, Mac) CCT3 (Huh7, MSC, U87, Mac) TCP1 (Huh7, MSC, U87, Mac)	psma2 (Huh7, MSC, U87, Mac) psma6 (Huh7, MSC, U87, Mac) psma4 (Huh7, MSC, U87, Mac) psma3 (Huh7, MSC, U87, Mac) psma1 (Huh7, MSC, U87, Mac) psma7 (Huh7, MSC, U87, Mac) psma5 (Huh7, MSC, U87, Mac)	psmb2 (Huh7, MSC, U87, Mac) psmb3 (Huh7, MSC, U87, Mac) psmb4 (Huh7, MSC, U87, Mac) psmb1 (Huh7, MSC, U87, Mac) psmb5 (Huh7, MSC, U87, Mac) psmb7 (MSC, U87) psmb6 (Huh7, MSC, U87, Mac)	psmc6 (Huh7, MSC, U87, Mac) psmc5 (Huh7, MSC, U87, Mac) psmc2 (Huh7, MSC, U87, Mac) psmc3 (Huh7, MSC, U87, Mac) ***psmc1*** (Huh7, MSC, U87, Mac) ***psmc4*** (Huh7, MSC, U87, Mac)
**V-ATPase**	**26S proteasome non-ATPase regulatory subunit**	**V-ATPase subunits not found in clastic cells**	**26S proteasome non-ATPase regulatory subunit not found in clastic cells**	**Arp2/3 complex**
A (Huh7, MSC, U87, Mac) B2 (Huh7, MSC, U87, Mac) E (Huh7, MSC, U87, Mac) H (Huh7, MSC, U87) ***AC45*** (Mac) C (Huh7, MSC, U87, Mac) ***d2*** d1 (Huh7, MSC, U87, Mac) PRR (MSC, U87, Mac) D (Huh7, MSC, U87, Mac) ***F*** G (U87)	psmd10 (MSC, U87, Mac) psmd14 (Huh7, MSC, U87, Mac) ***psmd7*** (Huh7, MSC, U87, Mac) psmd13 (Huh7, MSC, U87, Mac) psmd6 (Huh7, MSC, U87, Mac) psmd11 (Huh7, MSC, U87, Mac) psmd8 (Huh7, MSC, U87, Mac) psmd12 (Huh7, MSC, U87, Mac) psmd5 (Huh7, MSC, U87, Mac) psmd3 (Huh7, MSC, U87, Mac) psmd2 (Huh7, MSC, U87, Mac) psmd1 (Huh7, MSC, U87, Mac)	a1 (Huh7, MSC, U87, Mac) c (Mac)	psmd4 (Huh7, MSC, U87, Mac) psmd9 (U87)	arp3 (Huh7, MSC, U87, Mac) arp2 (Huh7, MSC, U87, Mac) p41 (Huh7, MSC, U87, Mac) p34 (Huh7, MSC, U87, Mac) p21 (Huh7, MSC, U87, Mac) p20 (Huh7, MSC, U87, Mac) p19 (Huh7, MSC, U87, Mac)

Parentheses after gene symbol indicates cell types where found: human monocyte-derived macrophages (Mac), U87 glioblastoma cells (U87), Huh7 hepatocellular carcinoma cells (Huh7) and human bone marrow-derived mesenchymal stem cells (MSC). Gene symbol in normal font indicates it is present in clasts, osteoclasts and odontoclasts. Gene symbol **in bold** indicates it is found in osteoclast and odontoclasts, but not clasts. Gene symbol underlined means it is found only in osteoclasts. Data from Huh7, MSC, U87 and Mac come from references [27,28].

### Protein complexes in clastic EVs

We detected elements of numerous stable protein complexes in clastic EVs. These included the actin-related 2/3 (Arp2/3) complex [30], the proteasome [31–33], the coatamer protein (COP) 1 complex [34], the T complex, the COP9 complex [35], and many, but not all subunits of the V-ATPase [36] (Tables 2 and S3). We also identified sets of proteins that are known to interact with each other transiently, including numerous proteins known to associate with the cytoskeleton at focal adhesions [37] and podosomes [38] and abundant glycolytic enzymes, which form complexes [39].

**Fig 5:**
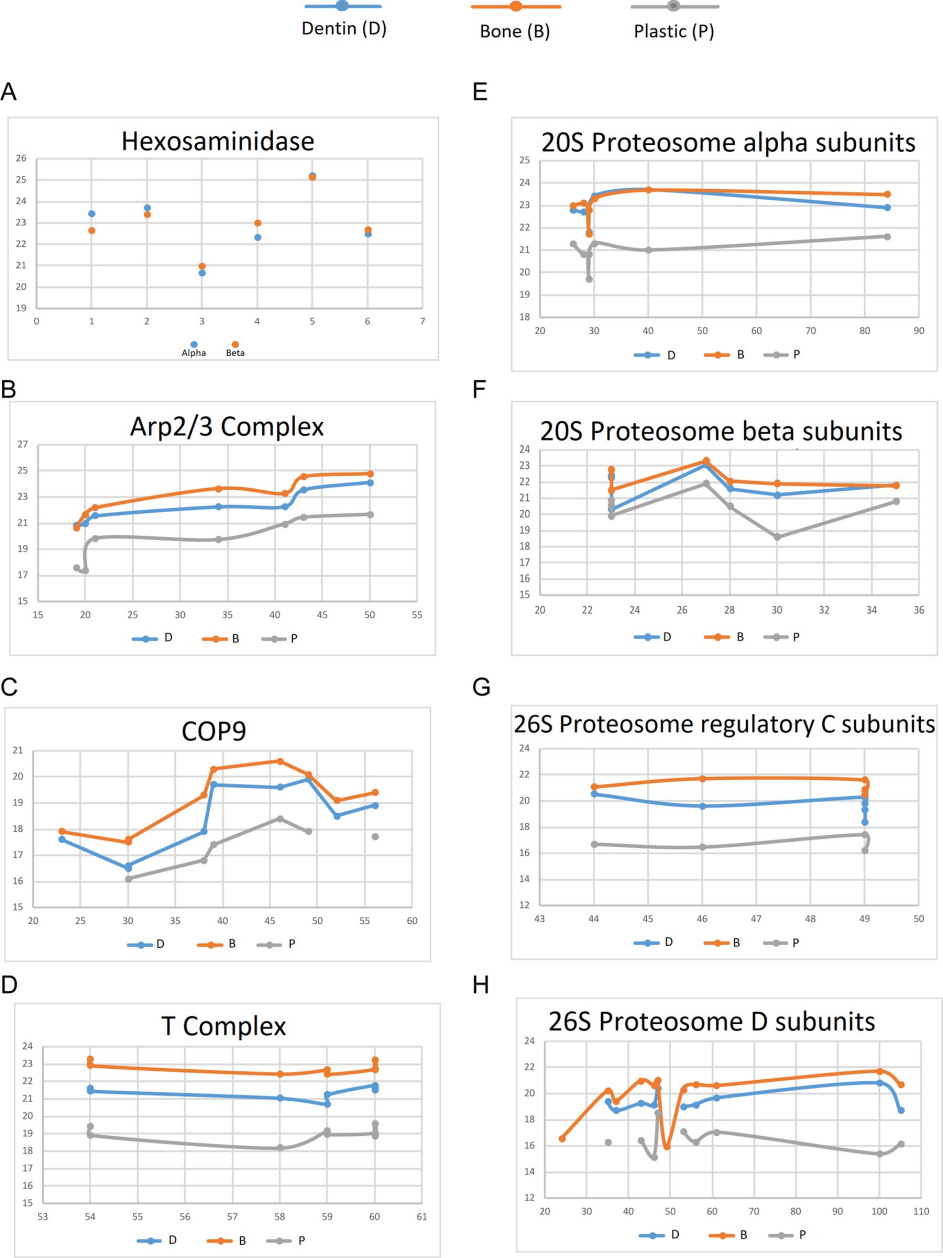
Evidence for protein complexes in clastic EVs. **(**A) Comparison of the relative abundance by 2D LC/MS/MS of hexaminidase alpha and beta, which are known to exist as a tight dimer across 6 experiments. (B-H) Various complexes found in clastic EVs, with relative abundance plotted against the expected amount of protein (molecular weight X expected stoichiometry). Note that the relative abundance estimate stays similar within all elements of a complex in a group; in most cases all elements of a complex in one group are similarly enriched or reduced compared to the other groups.

To determine the accuracy of the quantitative assessment of proteins we examined alpha and beta hexosaminadase. The two subunits form a stable dimer and the two proteins are similar in size. Assuming they enter EVs as a dimer, it would be expected that they would have similar abundance. As shown in Fig 5A, in six independent groups, the relative detected levels of alpha and beta hexosaminadase were very similar.

Several abundant complexes in clastic cell EVs with known protein sizes and stoichiometry were plotted by abundance, based on area under the slope of their peptides, against their molecular weight and expected stoichiometry (Fig 5B–5F). These data showed that the levels of proteins found in EVs was reasonably consistent with the levels predicted if they were elements of known protein complexes. These data also show that the relative level of the proteins in complexes varied coordinately between samples.

### A novel V-ATPase sub-complex is enriched in EVs from clastic cells

**Fig 6:**
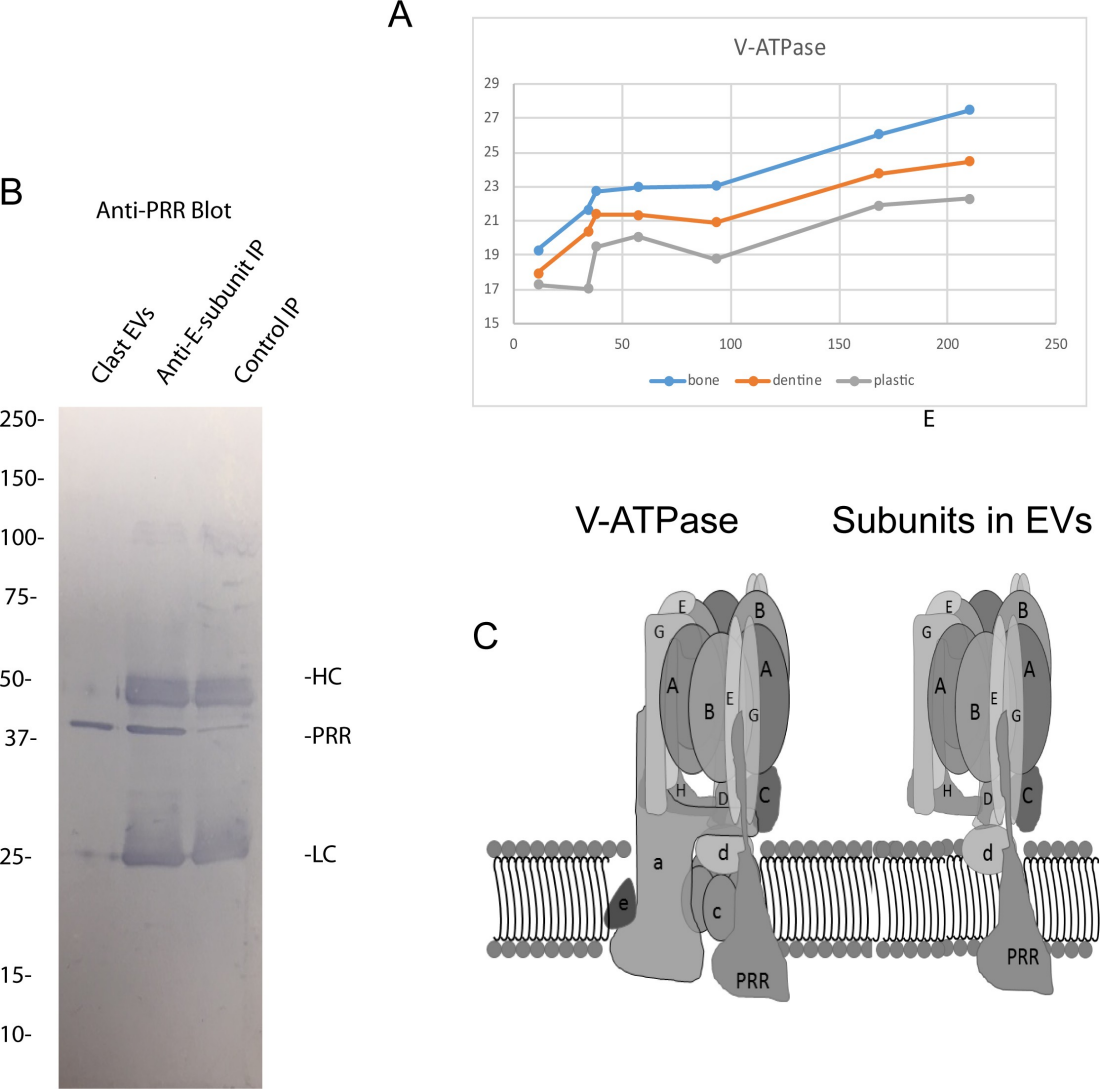
Evidence for a PRR-containing V-ATPase sub-complex in clastic EVs. (A) Relative abundance of the V-ATPase subunits and the PRR found in clastic EVs. (B) Left, Western blot of whole EVs stained with anti-PRR; right anti-E-subunit and IgG control immunoprecipitates (IP) from detergent-extracted EVs from clasts that were then blotted and probed with the anti-PRR antibody. HC and LC refer to the heavy and light chains of the antibodies used in the IPs. (C) Left is a schematic representation of the intact V-ATPase. The PRR has been positioned hypothetically in the intact enzyme. Right is a schematic of the subunits detected in osteoclast EVs assuming they are part of a single complex.

We focused attention on the V-ATPase subunits since they are very abundant in clastic cells compared with other cell types [40] and are vital for bone resorption [41]. As expected, V-ATPase subunits were among the most abundant elements of EVs from clastic cells (S3 Table). V-ATPases are rotary motors composed of 13 subunits: 8 peripheral proteins that make up the V1 complex, 3 integral proteins (a, b, and c) that form the proton pump channel [27], and 2 peripheral proteins (d and e). In addition, there are two accessory proteins, the (pro) renin receptor (PRR) [38] and AC45[39]. We detected all of the peripheral subunits of the V1 complex. These included the A-, B2-, C-, D-, E-, F-, G- and H-subunits. We also detected both isoforms of the d-subunit, d1 and d2. We did not detect the a-, b- or c-subunits that make up the proton channel. The PRR was very abundant, and we detected low levels of AC45 (S3 Table). The Z scores for the most abundant subunits were not significant between clastic groups (S2 Table). As with the other potential complexes, the relative expression pattern was similar in all samples (Fig 6A).

The B-subunit and C-subunit bind microfilaments [42–44], a possible means by which the peripheral V-ATPase subunits could be coordinately transported into EVs. However, the abundant PRR is a transmembrane protein that is vital to the assembly and/or function of V-ATPases [45]. We postulated that the peripheral V-ATPase subunits and d-subunits might be shuttled into EVs in association with the PRR. The PRR has been described in some cell types to be associated with the V-ATPase as a low molecular weight 8–9 kD cleaved protein [46]. The peptide coverage of PRR suggested that full length PRR is present in EVs and this was confirmed by Western Blot (Fig 6B). To test the idea that the PRR is in complex with other V-ATPase subunits in EVs, we solubilized EVs from clasts and immunoprecipitated using an antibody to the E-subunit. The resulting immunoprecipitate (IP) was then separated by SDS-PAGE, blotted, and probed with an anti-PRR antibodies. The PRR was detected in the anti-E subunit pulldown suggesting that it was in a complex that included E-subunit (Fig 6B). These data are consistent with the V-ATPase subunits present in EVs being in a membrane associated V-ATPase subcomplex that includes the PRR (Fig 6C).

## Discussion

In this report, we present the first high resolution 2D HPLC-MS/MS proteomic description of EVs released by clasts, osteoclasts and odontoclasts. Our data show that the substrate influences the protein composition of the EVs released from clastic cells, which all arise from the same hematopoietic precursors. In addition, our data suggest that not only the composition but also the quantity of EVs produced by clastic cells is a function of the differentiation state of the cells (resorbing vs non-resorbing) and the signal the cell is releasing from the substrate (dentin vs bone). A caveat is that the EVs may differentially bind to bone and dentin and therefore substrates may differentially deplete EVs, which help explain why non-resorbing clasts on plastic produced the highest average number of EVs. Previous studies report that RANK-containing EVs are crucial in intercellular regulation and may be used as a biomarker of bone resorption [1,4,29]. Interestingly, RANK was found in EVs from clasts and osteoclasts, but was not detected in EVs from odontoclasts (Fig 4). Both osteoclast and odontoclasts form specialized resorption apparatuses, composed of actin rings and ruffled membranes. Ruffled membranes are packed with V-ATPases [41,47,48]. V-ATPase subunits are major components of clastic EVs. Our findings are consistent with the results of our previous 1D analysis [6] and provide greater proteome coverage. Stabilin-1 and macrophage mannose receptor-1 were identified as abundant membrane proteins enriched in odontoclasts.

Overall, our proteomic data revealed that clastic EVs were enriched with proteins found in stable protein complexes (such as V-ATPase subunits, Arp2/3 complex and proteasome) or groups of interacting proteins (actin and actin-binding proteins [49], glycolytic enzymes [50]). The presence of these groups of proteins in fixed ratios, which corresponded to the proteins molecular weights and expected stoichiometry’s, could be explained by their being packaged into EVs as elements of super molecular assemblies. This finding is consistent with results of proteomic studies of EVs from other cell types. Indeed, a recent proteomic study of EVs from serum also reported several of the complexes that we detected in our study [51]. Packaging assembled protein complexes into EVs could serve as an efficient means to coordinately discard unneeded complexes. EVs could also shuttle pre-assembled protein complexes from cell to cell. A vital challenge for cells is to match protein production with the need to correctly assemble the proteins into super molecular assemblies [52,53]. Releasing surplus protein complexes in EVs to be taken up by other cells, might be of greater value than simply degrading them. This concept is consistent with the view of Takeuchi et al. [54] who previously proposed that EV-mediated transmission is a novel physiological mechanism to promote chaperone protein homeostasis at the organismal level.

We focused attention on the subunits of the V-ATPase that were detected in clastic EVs. Clastic EVs were enriched in what appears to be a sub-complex of V-ATPase subunits that includes the PRR. This interpretation was supported indirectly by the stoichiometry and directly by immunoprecipitation of the PRR using an antibody against the E-subunit. In contrast with clastic EVs, EVs from other cell types carry V-ATPase sub-complexes containing isoforms of the a-subunit, which is absent in the clastic EVs, but little or no PRR [27,28]. Clastic cells express 100-fold more V-ATPase than most other cell-types [40], and V-ATPase subunits were among the most abundant proteins found in clastic EVs. Since every V-ATPase sub-complex contains 10 or more proteins, V-ATPase subunits are in aggregate among the most abundant protein elements of EVs released by clastic cells.

In addition to being of interest as a novel V-ATPase sub-complex, and as potential biomarkers, the presence of full length PRR in EVs raises the possibility that these EVs may stimulate local renin/angiotensin signaling. The PRR binds pro-renin or renin and increases its ability to cleave angiotensinogen [55]. Recent studies indicate that bone remodeling is regulated in response to the renin/angiotensin signaling pathway [56–60]. Therefore, the PRR in EVs would be ideally located to activate (pro) renin or renin and regulate local renin/angiotensin signaling.

We identified an enrichment of stabilin-1 in EVs from resorbing cells, but not in EVs of non-resorbing clasts. Stabilin-1 is a large, multi-functional, type-1, transmembrane receptor that is found on tissue macrophages and serves as an immune regulator of inflammatory processes [61]. Other transmembrane proteins that were significantly enriched in EVs from odontoclasts compared with osteoclasts or clasts were macrophage mannose receptor-1 and CD36. As expected, we found RANK in EVs from clasts and osteoclasts [1,4,15,29]. However, it was not detected in EVs from odontoclasts. RANK-containing EVs were recently shown to be vital for coupling bone resorption and bone formation through reverse RANKL signaling [4].

In summary, we show that the protein composition of EVs released by osteoclasts, odontoclasts and non-resorbing clasts differed significantly. We conclude that the EV proteome was different among cell types and that the variability was due to the different matrix type and the resorbing state of the clastic cells. Due to their abundance in clastic EVs, we speculate that the V-ATPase subunits may be useful biomarkers of clast cell activity in liquid biopsy. In addition, we find clastic EVs to be enriched in a V-ATPase sub-complex that, to the best of our knowledge, has not been reported. Stabilin-1 and macrophage mannose receptor-1, abundant transmembrane proteins, were enriched in odontoclasts. Finally, RANK, which is involved in intercellular regulation by osteoclast EVs [1,4], was found in clastic EVs at levels consistent with previous reports.

## Supporting information

S1 Table Proteins present in extracellular vesicles (EVs) of osteoclasts, odontoclasts and non-resorbing clasts. Proteins and peptides are sorted by their mass spectrometry signal intensities and number of peptide hits for identification.
(XLSX)

S2 Table Pairwise comparisons of proteins present in extracellular vesicles (EVs) and their respective Z-scores. (XLSX)

S3 Table Protein complexes in extracellular vesicles (EVs) and their respective mass spectrometry signal intensities. (XLSX)
